# Group A Streptococcal M1 Protein Provides Resistance against the Antimicrobial Activity of Histones

**DOI:** 10.1038/srep43039

**Published:** 2017-02-21

**Authors:** Simon Döhrmann, Christopher N. LaRock, Ericka L. Anderson, Jason N. Cole, Brinda Ryali, Chelsea Stewart, Poochit Nonejuie, Joe Pogliano, Ross Corriden, Partho Ghosh, Victor Nizet

**Affiliations:** 1 Department of Pediatrics, University of California San Diego, La Jolla, California, United States of America; 2 Department of Chemistry and Biochemistry, University of California San Diego, La Jolla, California, United States of America; 3 Department of Biological Sciences, University of California San Diego, La Jolla, California, United States of America; 4 Department of Pharmacology, University of California San Diego, La Jolla, California, United States of America; 5 Department of Skaggs School of Pharmacy and Pharmaceutical Sciences, University of California San Diego, La Jolla, California, United States of America; 6 Rady Children’s Hospital, San Diego, California, United States of America

## Abstract

Histones are essential elements of chromatin structure and gene regulation in eukaryotes. An unexpected attribute of these nuclear proteins is their antimicrobial activity. A framework for histone release and function in host defense *in vivo* was revealed with the discovery of neutrophil extracellular traps, a specialized cell death process in which DNA-based structures containing histones are extruded to ensnare and kill bacteria. Investigating the susceptibility of various Gram-positive pathogens to histones, we found high-level resistance by one leading human pathogen, group A *Streptococcus* (GAS). A screen of isogenic mutants revealed that the highly surface-expressed M1 protein, a classical GAS virulence factor, was required for high-level histone resistance. Biochemical and microscopic analyses revealed that the N-terminal domain of M1 protein binds and inactivates histones before they reach their cell wall target of action. This finding illustrates a new pathogenic function for this classic GAS virulence factor, and highlights a potential innate immune evasion strategy that may be employed by other bacterial pathogens.

In 1884, Albrecht Kossel discovered that DNA inside the nucleus is associated with proteins, naming these proteins histones^1^. Subsequently, several histone isoforms have been discovered - the linker histone H1 and the core histones H2A, H2B, H3 and H4^2^. Histones regulate DNA packaging and gene expression as first hypothesized by Stedman and Stedman in 1950^2^ and later proven in numerous studies of post-translational histone modification^3^. In 1958, Hirsch found that purified histones exhibit antibacterial activity^4^, a finding later corroborated by many other investigators^5^,^6^,^7^, with unclear implications in innate immune defense.

In 2004, the Zychlinsky group discovered a novel function of neutrophils termed neutrophil extracellular traps (NETs), wherein neutrophils extrude their DNA to forms a lattice network capable of ensnaring bacteria and exposing them to a high concentration of antimicrobial peptides and proteases^8^. NET generation or “NETosis” is stimulated upon infection with a variety of Gram-negative and Gram-positive bacteria and their secreted exotoxins^8^,^9^,^10^,^11^,^12^,^13^, as confirmed by direct visualization *in vivo*^14^. The process of NETosis elaborates abundant quantities of histones into the extracellular milieu, estimated at 2.5 mg histones per 10^9^ neutrophils, such that histones comprise 70% of the protein content within the NET architecture^15^. Besides NETs, other myeloid cell lineages including mast cells^16^, macrophages^17^, basophils^18^ and eosinophils^19^ can also deploy histones within DNA-based extracellular traps (ETs). In addition to proposed antimicrobial properties, released histones can induce chemokine production to augment leukocyte recruitment^20^.

Group A *Streptococcus* (GAS) is an important human pathogen responsible for over 700 million infections annually, ranking among the top 10 causes of infection-associated mortality worldwide^21^. GAS can produce a wide spectrum of infections ranging from pharyngitis (“strep throat”) to invasive conditions including necrotizing fasciitis (“flesh-eating disease”) and streptococcal toxic shock syndrome^22^. The ability of GAS to spread in the bloodstream and cause systemic infection bespeaks a multifaceted resistance to clearance by neutrophils, which provide a critical first line of host defense against bacterial invasion^23^,^24^,^25^. Here, we investigated the role of neutrophil-derived extracellular histones in defense against Gram-positive pathogens.

In this study, we found a robust release of histones by neutrophils exposed to several medically important Gram-positive pathogens. GAS exhibited a remarkable resistance to histone killing, with a minimum inhibitory concentration (MIC) of 125–250 μg/mL. Mutant screening and loss- and gain-of-function analysis identified the classical virulence factor M1 protein as necessary and sufficient for high-level histone resistance compared to other species. Mutational and biochemical analyses further reveal that M1 protein acts through a mechanism of direct binding and inactivation.

## Results

### Histones are released from neutrophils in response to PMA or bacteria

**Figure 1:**
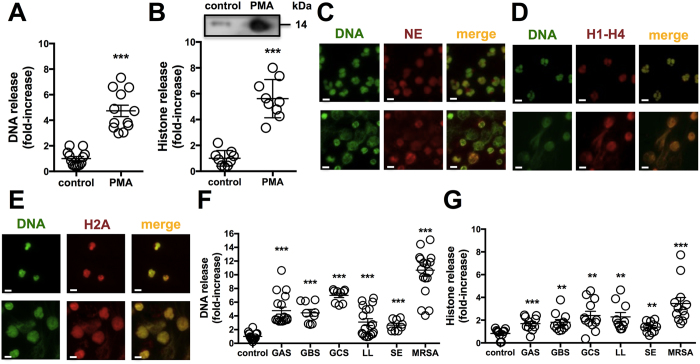
Histones are released from neutrophils. Released (**A**) DNA was quantified by staining with the cell-impermeable dye Sytox Green or (**B**) histones from neutrophils after 4 h stimulation with 25 nM PMA as compared to controls via IHC fluorescent measurements using primary rabbit anti-histone H2A antibody and secondary goat anti-rabbit Alexa 488 antibody. NETs were induced by stimulation with 25 nM PMA for 4 h as analyzed by immunofluorescence (IF) microscopy using primary (**C**) anti-neutrophil elastase (NE), (**D**) anti-histone H1-H4 and (**E**) anti-histone H2A antibody followed by secondary Alexa 594 antibody (red) and Sytox Green to stain DNA (green). (**F**) DNA release from neutrophils infected with a panel of six different Gram-positive bacteria at an MOI of 10 was determined by incubation with cell-impermeable Sytox Green at 4 h. (**G**) Histone release in response to bacteria at MOI of 10 was detected by IHC using Alexa 488 antibody against polyclonal anti-histone H2A antibody at a 4 h time point. For (**C–E**) randomized images with scale bars representing 10 μm are shown and are representative of at least three independent experiments. Experiments in (**A,B** and **F,G**) were combined from a minimum of three independent experiments in triplicates and normalized to untreated control at each experiment. Results shown represent average ± SEM values and were analyzed by Student’s t-test (**P < 0.01, ***P < 0.001).

Neutrophils actively migrate as “first responders” to sites of infection and deploy several antimicrobial effector mechanisms, including NETs, to contain and kill invading pathogens^8^. Neutrophils were stimulated with phorbol 12-myristate 13-acetate (PMA), a protein kinase C (PKC) agonist and canonical stimulator of NETs, for 4 h and we found a robust release of DNA by using the cell-impermeable, fluorescent DNA dye Sytox Green (Fig. 1A) and histones coupled to immunohistochemistry (IHC) for pooled histones (H1-H4) or western immunoblot for histone H2A (Fig. 1B). We calculated the closely coupled release of DNA and histones to be increased 5 to 6-fold in PMA-stimulated neutrophils compared to untreated control cells. At 4 h post-PMA stimulation, neutrophil elastase (NE) (Fig. 1C), histone mixture (H1-H4) (Fig. 1D) and histone H2A (Fig. 1E) all co-localized with extracellular DNA in NETs. Exposure of neutrophils to representative strains of Gram-positive bacteria GAS, group B *Streptococcus* (GBS), group C *Streptococcus* (GCS), *Lactococcus lactis* (LL), *Staphylococcus epidermidis*, and methicillin-resistant *Staphylococcus aureus* (MRSA) at multiplicity of infection (MOI) = 10 bacteria per neutrophil, stimulated significant release of DNA (Fig. 1F) and histones (Fig. 1G) within 4 h, with the MRSA strain appearing the most potent inducer, consistent with a previous report on MRSA as a rapid trigger for NETs^26^. These data establish an abundant release of histones within NETs in response to pharmacological activation or bacterial exposure^8^,^15^.

### GAS is resistant to histones and NETs

**Figure 2:**
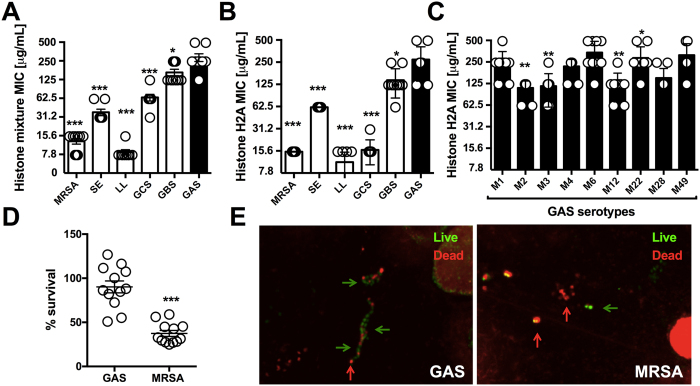
Antimicrobial activity of histones against Gram-positive bacteria. The resistance of multiple Gram-positive bacterial species was tested in MIC assays against (**A**) histone mixture and (**B**) histone H2A. (**C**) Resistance of representative GAS M serotypes 1, 2, 3, 4, 6, 12, 22, 28 and 49 against histone H2A in MIC assay were performed. (**D**) NET-mediated killing of GAS WT and MRSA WT was performed at an MOI of 0.1 at 15 min post-infection and bacterial survival was calculated by CFU enumeration vs. initial inoculum. (**E**) Representative confocal microscopy image of NET-mediated killing for GAS and MRSA at an MOI of 1 by Live/Dead BacLight staining with indication to identify dead (red arrows) and live (green arrows) bacteria. Results shown represent average ± SEM values and were analyzed by Student’s t-test (**D**) or Mann-Whitney test (**A–C**) (*P < 0.05, **P < 0.01, ***P < 0.001). Each dot in (**A–D**) represents one sample and all experiments have been performed at least three independent experiments in triplicates.

Tying the classical estimation of histone antimicrobial potential by Hirsch^4^ and others with the abundant histone content in NETs in response to live bacterial challenge, we next examined the relative sensitivity of various Gram-positive bacterial species to pooled histones in a MIC assay. We found GAS to be the most resistant bacteria to killing by pooled histones with an MIC of 250 μg/mL (Fig. 2A); no surviving bacteria were identified upon sample plating onto antibiotic-free media at the experimental endpoint (lower limit of detection = 200 colony forming units (CFU)/ml indicating >99.9% reduction in CFU), indicating that histones exhibit bactericidal activity and the minimal bactericidal concentration (MBC) values matched the corresponding MIC values for all species. For comparison, human cells were much more sensitive to histone toxicity than GAS, with primary neutrophils and cultured A549 lung epithelial cells exhibiting 30% to 70% cytolytic cell death, respectively, following 1 h exposure to 1000 μg/mL histone mix (Figure S1). Histone H2A is the most abundant histone species present in NETs^15^, and the MIC/MBC values for all bacteria to purified Histone H2A was similar to that for pooled histone, with GAS again exhibiting high-level resistance (MIC/MBC = 250 μg/mL) (Fig. 2B). MIC testing of nine different GAS strains of multiple M serotypes (1, 2, 3, 4, 6, 12, 22, 28 and 49) associated with invasive human infections showing high-level histone resistance was broadly shared among all isolates ranging between MIC values of 125–500 μg/mL (Fig. 2C). To probe for functional histone resistance against the biological framework of their extracellular release, we induced NETs with PMA for 4 h and incubated the formed NETs with bacteria. Enumeration of surviving colony forming units (CFU) at 15 min (Fig. 2D) or confocal fluorescent microscopy for live (green) vs. dead (red) bacteria staining (Fig. 2E) showed that GAS was not killed efficiently by NETs, whereas MRSA was. Thus GAS is highly resistant to purified histones and to NETs, which represent the predominant mechanism of histone release during bacterial infection.

### M1 protein from GAS protects against histones

**Figure 3:**
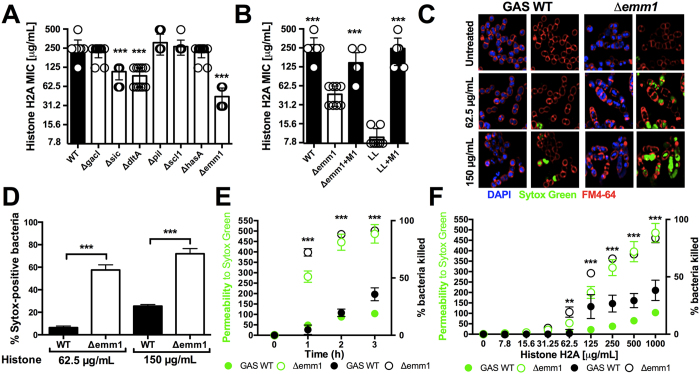
GAS M1 protein protects against histones. (**A**) Screening of virulence-associated genes with GAS M1 background of isogenic mutants Δ*sic*, Δ*dltA*, Δ*gacI*, Δ*hasA*, Δ*scl1*, Δ*pil* and Δ*emm1* mutants compared to GAS M1 WT. (**B**) Histone H2A MIC assays with GAS WT, Δ*emm1* mutant and complemented mutant (Δ*emm1*+pM1) as well as LL and heterologous expression of M1 protein in LL (LL+pM1) are shown. (**C**) Effect of histone treatment on GAS WT and Δ*emm1* mutant bacteria were visualized using the cell-impermeable DNA dye Sytox Green (green), the cell-permeable DNA dye DAPI (blue) and the membrane dye FM4-64 (red) by confocal microscopy after 3 h of incubation in the presence or absence of histone at 62.5* *μg/mL or 150* *μg/mL histone H2A and (**D**) the Sytox Green-positive population in random view fields was quantified. (**E**) Time-dependency of permeability was assessed using the cell-impermeable dye Sytox Green, which yielded into fluorescent signal upon interaction of dye with bacterial DNA, and was monitored with 1000* *μg/mL histone for GAS WT and Δ*emm1* mutant and bacterial survival was simultaneously calculated via CFU enumeration relative to initial inoculum over a 3 h time course. **(F)** Bacterial permeability was investigated by treatment with increasing concentrations of histones ranging from 7.8* *μg/mL to 1000* *μg/mL histones and simultaneous CFU enumeration of bacterial survival relative to initial inoculum after 3 h incubation. Results shown represent average ± SEM values and were analyzed by Student’s t-test in (**D–H**) or Mann-Whitney test in (**A,B**) (**P < 0.01, ***P < 0.001). Each dot represents one sample. All experiments have been carried out a minimum of three times run in triplicates, except for (**C**) which was repeated three times in monoplicates.

Screening multiple isogenic mutants lacking individual candidate virulence factors in virulent GAS serotype M1 strain 5448, we found that loss of *sic* (streptococcal inhibitor of complement), *dltA* (encoding lipoteichoic acid D-alanylation) and *emm1* (encoding the surface-anchored M1 protein) genes contribute to histone resistance (Fig. 3A). The strongest increase in histone sensitivity, approximately 4- to 8-fold, was observed in the GAS Δ*emm1* mutant. The M protein, which is very abundantly expressed on the GAS surface, is the best-studied, classical virulence factor of the pathogen contributing to antigenic diversity, cellular invasion, complement and antimicrobial peptide resistance, impairment of phagocytosis and inflammatory activation^27^,^28^,^29^,^30^,^31^, but has not previously been implicated to conferring resistance against the antimicrobial effect of histones. Complementation of the GAS Δ*emm1* mutant with plasmid-borne *emm1* (pM1) restored high-level resistance to histones, while heterologous expression of M1 protein in LL conferred high-level resistance in a gain-of-function analysis (Fig. 3B). Resistance of the GAS WT and Δ*emm1* mutant in MIC experiments to pooled histone mix paralleled findings with purified histone H2A (Figure S2).

To further characterize histone protection mediated via the GAS M1 protein, we performed confocal microscopy using the cell-permeable DNA stain DAPI (blue), the cell-impermeable DNA stain Sytox Green (green), and the bacteria cell membrane stain FM4–64 (red). After 3 h of incubation in the presence of histone H2A (62.5 or 150 μg/mL), Δ*emm1* mutant bacteria showed a large increase in intracellular Sytox Green signal indicating disruption of the cell wall and bacterial death compared to the WT GAS parental strain (Fig. 3C,D). In a plate-based adaptation of the assay, histone H2A produced marked dose-dependent (over 0–1000 μg/mL range) and time dependent (1–3 h at 1000 μg/mL) increases in Sytox Green signal in the Δ*emm1* mutant compared to WT GAS, correlating with the percentage of killed bacteria by CFU enumeration (Fig. 3E,F). These data show that M1 protein protects GAS against histone-mediated disruption of the bacterial cell wall.

### M1 protein protects against released, extracellular histones in NETs

**Figure 4:**
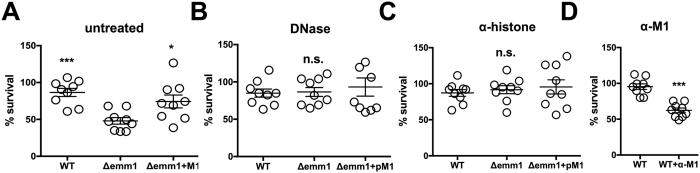
M1 protein protects against histones in NETs. 2 × 10^5^ neutrophils were stimulated for 4 h with 25 nM PMA to induce NETs and infected with GAS WT, Δ*emm1* mutant and complemented strain (Δ*emm1*+pM1) at an MOI of 0.1. Surviving CFUs were calculated relative to initial inoculum after 15 min of infection in (**A**) untreated cells, (**B**) cells treated with 100 U/mL DNase 1 to degrade the DNA backbone, or (**C**) cells treated with blocking anti-histone H2A antibodies. (**D**) Survival in NETs of GAS WT naïve or anti-M1 antiserum at MOI of 0.1 after 15 min of infection. All results are showing the percentage of bacterial survival relative to the bacterial inoculum. Each dot represents one sample from three independent experiments in triplicates and neutrophils were obtained from three different donors. Results shown represent average ± SEM values and were analyzed by Student’s t-test (n.s., not significant and *P < 0.05, ***P < 0.001).

The M1 protein plays a protective role in resistance to NET killing^31^,^32^,^33^. Whether this protective effect is attributable all or in part to conferred resistance against histones is not known. We used an established method that recovers bacteria ensnared in NETs by vigorous trituration with Triton X-100, allowing us to distinguish CFU changes as bacterial killing rather than simple clumping^34^. As a proof of concept, we activated neutrophils with PMA for 4 h to maximize NET production and then infected them with GAS M1 WT, Δ*emm1* mutant and the complemented strain (Δ*emm1*+pM1) at MOI = 0.1 for 15 min. The Δ*emm1* mutant was highly susceptible to killing by NETs compared to the WT or complemented strain (Fig. 4A). Degradation of the DNA backbone of NETs with DNase (Figure S3) restored survival of the GAS Δ*emm1* mutant to the level of the WT and complemented strain (Fig. 4B), indicating that entrapment of the bacteria, and consequent exposure to high local antimicrobial concentration, is necessary for GAS Δ*emm1* killing within NETs. Using blocking anti-histone antibodies, we found that survival of the GAS Δ*emm1* mutant was restored to the level of the GAS WT and complemented strain (Fig. 4C); these findings suggest that despite NETs containing numerous antimicrobial factors acting in concert, the M1 protein primarily protects against histones. WT GAS survival in NETs was likewise significantly reduced with serum containing neutralizing antibodies raised against recombinant M1 protein, but not in the presence of serum from naive mice (Fig. 4D), further suggesting that protection against histones requires a functional interaction of M1 protein and histones.

### M1 protein directly binds histones

**Figure 5:**
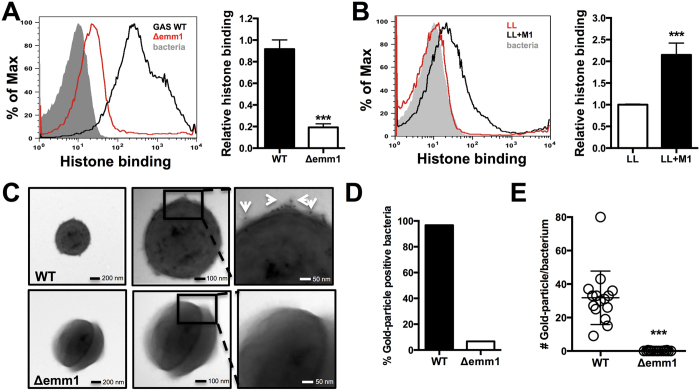
M1 protein binds to and sequesters histones. (**A**) Binding of histone H2A at 31.25* *μg/mL to whole, live GAS M1 WT and Δ*emm1* mutant bacteria was determined via IHC using primary anti-histone H2A antibody followed by secondary Alexa 488 antibody by shift in fluorescence intensity by flow cytometry and quantified using the geometric mean fluorescence intensity (gMFI). (**B**) Binding of histone H2A was analyzed for whole, live LL WT and LL+pM1 bacteria by flow cytometry and quantified by gMFI. Visualization of histone binding to the most surface-exposed protein from GAS, the M1 protein (hair-like structures) of GAS M1 WT and Δ*emm1* mutant (**C**) using primary rabbit anti-histone H2A antibodies followed by secondary anti-rabbit immunogold-labeled antibodies visualizing 15 nm gold particle with white arrows by transmission electron microscopy (TEM). (**D**) Qualitative binding of histones as determined by identification of immune-gold positive bacteria and quantification of immune-gold particle number per bacterium were determined from > 20 bacteria in random view fields at a 49,000x magnification. Results in (**A–C**) show representative results or TEM images from at least three independent experiments. Quantification of results obtained by flow cytometry in (**A,B,E**) represent average ± SEM values and were analyzed by Student’s t-test (***P < 0.001).

M protein is the most abundant protein on the surface of GAS^35^ and forms hair-like fimbriae extending from the bacterial surface^36^, as we verified by transmission electron microscopy (TEM) and flow cytometry analysis in our WT GAS model strain 5448 (Figure S4). We analyzed binding of histones to live WT GAS and Δ*emm1* mutant bacteria by flow cytometry, and found a 4-fold reduction in histone binding to the M1-protein deficient strain (Fig. 5A). Similarly, heterologous expression of the M1 protein in LL increased histone binding to live bacteria by more than 2-fold in the same assay (Fig. 5B). Immunogold TEM analysis using anti-histone antibodies showed histones bound to the WT GAS strain, but not the Δ*emm1* mutant (Fig. 5C). Our qualitative analysis showed that almost all analyzed WT GAS bacteria were positive for gold-particles indicating bound histones compared to less than 10% of Δ*emm1* mutant bacteria (Fig. 5D), with WT binding on average >30 gold-particles per bacterium in contrast to mutant bacteria with almost no detection (Fig. 5E). These results suggest that histone protection occurs via a scavenging and inactivation mechanism mediated by M1 protein, preventing histones from penetrating to the underlying bacterial cell wall.

### The M1 protein N-terminus binds histones to mediate histone resistance

**Figure 6:**
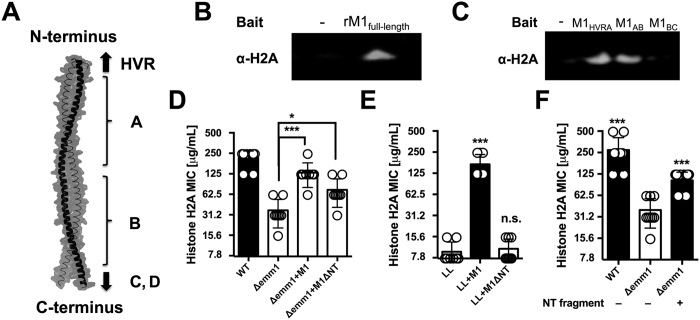
N-terminal portion of M1 protein binds to histones and mediates resistance against histones. (**A**) Schematic of M1 protein (2OTO) model highlighting the N-terminal hyper-variable region (HVR), and A and B repeat and the start of the C and D repeat which terminate at the C-terminal cell wall anchor. (**B**) Full length, recombinant M1 protein (rM1full-length) was used as bait to analyze interaction with histone H2A as determined by pull-down analysis and developed by western blot. (**C**) Binding of recombinant, truncated rM1 fragments HVR+A, A+B or B+C part of M1 protein to histone H2A by pull-down was visualized by western blot. (**D**) GAS M1 WT, Δ*emm1* mutant, Δ*emm1*+pM1 and a complemented strain lacking the NT region (Δemm1+pM1ΔNT) were tested for resistance to histone H2A by MIC as well as (**E**) LL WT, LL+pM1 and LL+pM1ΔNT. (**F**) Effect of exogenous 10 μM NT fragment on histone killing for GAS Δ*emm1* mutant in MIC testing. Results shown were obtained from at least three independent experiments. Results shown represent average ± SEM values and were analyzed by Mann-Whitney test in (**D–F**) (*P < 0.05, ***P < 0.001).

The M1 protein forms a dimeric, α-helical, coiled-coil structure^37^ extending approximately 50 nm from the GAS cell wall^38^. The M1 protein consists of a hyper-variable region (HVR) at the N-terminus, followed by repeats of A and B regions and the conserved C and D regions at the C-terminus, which is anchored to the cell wall via LPXTG motif 37 as shown schematically in Fig. 6A. We used recombinant full-length M1 protein (rM1) and M1 protein fragments to analyze direct interactions with histone H2A. Full-length rM1 bound H2A in a pull-down assay as revealed by western blot (Fig. 6B); this interaction held for M1 protein fragments containing the HVR and A region as well as the A and B region, but not a fragment containing the B region and one of the repeats of the C region (Fig. 6C). Thus, we identified the N-terminal (NT) and most surface-exposed part of the recombinant M1 protein (rNT) containing the HVR, A and B regions to mediate binding to histones. Upon complementation of the GAS Δ*emm1* mutant, or heterologous expression in WT LL, a truncated version of the *emm1* gene lacking a large portion of the NT-terminal region (designated M1_ΔNT_) failed to confer histone resistance (Fig. 6D,E), corroborating that the NT fragment of the M1 protein is necessary for histone protection. Furthermore, exogenous addition of 10 μM recombinant NT fragment (rNT) to MIC assays with GAS Δ*emm1* mutant resulted in a 2-fold increase in resistance to histones in MIC testing (Fig. 6F). Taken together, our data demonstrate that the NT domain of the GAS M1 protein confers resistance to histones through a binding and inactivation mechanism, allowing the pathogen to tolerate high concentrations of histones and promoting survival in NETs.

## Discussion

While a broad-spectrum antimicrobial activity of purified histones was discovered by Hirsch in 1958^4^ and subsequently many others^5^,^6^,^7^, it was not until the discovery of NETs in 2004 that a conceptual framework for the biological relevance of extracellular histones as antimicrobials was revealed. Because histone knockout animals are non-viable, the role of histones in innate immune defense *in vivo* is limited to inference from mechanisms leading to the release of histone, such as in NETs^13^. The aim of this study was to assess the direct antimicrobial role for histones in innate immune defense against Gram-positive bacteria. Of note, a beneficial role for histones in recruiting innate immune cells to the site of infection^20^ and a pathological role for histones in sepsis^39^ have recently been reported.

Specific histone resistance factors have been described for the model Gram-negative bacterium *Escherichia coli* including the O-antigen from lipopolysaccharide (LPS)^4^,^40^,^41^ and outer membrane protease T (OmpT)^42^. In histone resistance assays of Gram-positive bacteria, we observed a remarkably high resistance of GAS compared to other species examined. This phenotype was conserved across several different GAS serotypes and reminiscent of Hirsch’s original study, in which GAS was resistant to the highest concentration of histones tested^4^. We also demonstrated that the high resistance of GAS to histones directly translated into high resistance to NETs, which is consistent with findings that histones are not only a structural component of NETs, but also a major bactericidal factor in these structures^8^,^15^. Of note, histone release has been documented in mouse models of GAS infection and in tissue biopsies of human patients suffering from cutaneous and invasive GAS infections^20^,^43^.

Because the common anionic bacterial cell wall component lipoteichoic acid (LTA) is known to bind cationic histones^44^,^45^ and would be present in all species examined, we hypothesized that other unique factor(s) from GAS must interact with histones to mediate the high-level resistance. Additional mechanisms can contribute this activity, as a recent study found an M49 strain of GAS to be capable of hijacking host plasmin to degrade histones^46^. In our study of the hyper-invasive M1T1 GAS strain, we identified the surface expressed M1 protein, a multifunctional virulence factor^28^,^37^,^38^, as responsible for this high-level histone resistance. Despite enhanced resistance to their activity, flow cytometry and TEM analysis at sub-MIC concentrations show that the WT GAS strain binds histones more strongly than an isogenic Δ*emm1* mutant. This counter-intuitive relationship suggests that M1 protein “traps” or “scavenges” histones to prevent them from reaching their cell wall target of action, in contrast to *dltA*-mediated resistance (Fig. 3A), where incorporation of cationic residues into the bacterial cell wall likely decreases histone attraction. Histone-binding via M1 protein was mapped to the most surface-exposed NT domain of M1 protein, which was required for functional complementation of the Δ*emm1* mutant, and conferred passive protection against histone killing when provided in soluble, recombinant form.

Our studies with purified histones correlate with higher resistance of WT GAS compared to Δ*emm1* mutant against clearance in NETs *ex vivo*^31^,^32^, but here we demonstrate that this phenotype is predominantly dependent on histone action over other NET-associated antimicrobials such as cathelicidin peptide LL-37^32^. M1 protein is an important resistance mechanism to histone killing and further highlights the role for these ancient and potent host defense proteins as a component of innate immunity against invasive infection.

## Materials and Methods

### Bacterial Strains

GAS wild-type (WT) strain M1T1 5448 was originally isolated from a patient with necrotizing fasciitis and toxic shock syndrome^47^. GAS WT, Δ*emm1* mutant and plasmid complementation (Δ*emm1*+pM1) strains and *L. lactis* NZ9000 WT (LL) heterologously expressing *emm1* gene on a plasmid (LL+pM1), were previously described^32^. Multiple isogenic mutants Δ*sic*, Δ*dltA*, Δ*scl1*, Δ*hasA*, Δ*gacI* and Δ*pil* in the GAS M1 5448 background, a panel of nine GAS isolates of the M serotypes (1, 2, 3, 4, 6, 12, 22, 28 and 49) and strains of methicillin-resistant *Staphylococcus aureus* TCH1516 WT (MRSA), *Staphylococcus epidermidis* ATCC 1457 WT (SE), group B *Streptococcus* COH1 WT (GBS) and group C *Streptococcus* 10565 WT (GCS) were tested. Bacteria were cultivated in Todd-Hewitt broth (THB) at 37 °C, supplemented with 50 μg/mL erythromycin in THB for heterologous expression constructs and for MRSA and *S. epidermidis* at 37 °C with shaking at 200 rpm.

### Isolation of human neutrophils

Neutrophils were isolated from healthy donors using PolyMorphPrep (Fresenius Kabi, Oslo, Norway) as previously described^48^, following a protocol approved by the University of California San Diego Human Research Protections Program. All donors provided informed consent, and all methods were carried out in accordance with relevant guidelines and regulations. Viability was assessed using 0.04% trypan blue staining by microscopy.

### Induction and visualization of NETs

NET induction assays were performed as previously described^49^. Briefly, 2 × 10^5^ neutrophils were seeded into a 96-well plate and infected with log-phase bacteria at an MOI of 10. As positive control neutrophils were stimulated with 25 nM PMA or as negative controls left untreated or PMA-activated neutrophils combined with deoxyribonuclease I (DNase). Cells were incubated for 4 h at 37 °C/5% CO_2_ and DNA release was determined by using the cell-impermeable DNA-staining dye Sytox Green at 5 μM. The fluorescence signal was quantified at 485 nm ex/527 nm em.

NET visualization by immunofluorescence microscopy (IF) was performed as described previously^49^. Briefly, 1 × 10^5^ neutrophils, either unstimulated or activated with 25 nM PMA, were incubated for 4 h. After stimulation, cells were fixed with 4% paraformaldehyde (PFA), blocked with Dulbecco’s buffered-phosphate saline (DPBS) +2% bovine serum albumin (BSA) +2% serum and subsequently stained with primary anti-H2A antibody (1:200 dilution), antiserum against histone mixture (this study) or anti-neutrophil elastase antibody (NE) in DPBS+2% BSA overnight at 4 °C followed by incubation with secondary Alexa 594 antibody (1:500 dilution) for 1 h at room temperature (RT). DNA was stained with Sytox Green. NETs were imaged on a fluorescent microscope at 40x magnification. Representative, randomized images are shown.

### Histone release from neutrophils

Neutrophils at 2 × 10^5^ were untreated or activated with 25 nM PMA and incubated for 4 h in 96 well-plate tissue culture-treated. The supernatant was carefully removed from cells in non-permeabilizing conditions. Cells were then blocked with Hanks’ balanced salt solution (HBSS) with Ca^2+^ and Mg^2+^ with 10% BSA for 30 min and incubated with anti-H2A antibody (1:500 dilution) in HBSS +1% BSA for 30 min at RT. A final incubation with secondary Alexa 488 antibody (1:500 dilution) in HBSS+2% BSA was carried out for 30 min. After washing, extracellular histones released from neutrophils were quantified at 488 nm ex/538 nm em. Controls included no cells as background and DNase treatment of PMA-activated neutrophils.

### Western blotting

A total of 1 × 10^7^ neutrophils were either untreated or activated with 25 nM PMA and were incubated for 4 h. After 3.5 h, neutrophils were treated with 100 U/mL DNase for 30 min. Proteins were then separated from cells by centrifugation at 12,300 × g for 5 min and proteins in the supernatant were collected, precipitated with 15% trichloroacetic acid (TCA) and incubated overnight at 4 °C. Proteins were washed twice with ice-cold acetone and denatured in SDS sample buffer. Proteins were separated on a 12% SDS page gel and transferred onto 0.2 μM PVDF membrane. The membrane was developed with anti-histone H2A antibodies (1:200 dilution) in DPBS+2% BSA with 0.1% Tween-20 and incubated for 1 h at RT followed by detection with secondary IRDye 800 CW conjugated antibody (1:500 dilution) for 1 h. Membranes were then washed with DPBS and imaged with Odyssey LI-COR.

### NET-mediated killing of bacteria

NET-mediated killing experiments were performed as previously described^34^. Briefly, 2 × 10^5^ neutrophils were stimulated with 25 nM PMA for 4 h to induce NETs. Log-phase bacteria were washed once to remove secreted nucleases and added to neutrophils at an MOI of 0.1 for 15 min. As controls, NETs were degraded with DNase, the bactericidal activity of histone H2A was blocked prior to infection with 3 μg/mL anti-histone H2A antibody or the M1 protein from GAS WT was blocked with polyclonal anti-M1 serum (in-house) against recombinant M1 protein or naïve serum as control at 1:200 dilution for 30 min and was washed once prior to challenge. CFU was enumerated by plating onto THA after vigorous trituration with 0.025% Triton X-100 to free bacteria from NETs and prevent clumping. Bacterial survival was calculated as percentage of the initial inoculum.

### Bacterial viability analysis by microscopy

NETs were induced with 25 nM PMA for 3.5 h in 8-well LabTek II slides, followed by infection of MRSA and GAS at an MOI of 1 as described previously^34^. After 20 min, cells were stained using Live/Dead BacLight Viability Kit according to manufacturer’s instructions. Samples were fixed with 4% PFA and imaged at 63x magnification using a confocal microscope.

### Histone MIC assay

Histone assays were performed with histone H2A or mixture from calf thymus. Log-phase bacteria at 2 × 10^5^ CFU were incubated with serial 2-fold diluted histones starting at 500 μg/mL to 7.8 μg/mL in RPMI-1640 with phenol red (RPMI) plus 5% THB at 37 °C for 24 h for MIC analysis. The rNT fragment, representing residues 42–194 of M1 protein, was exogenously added at a concentration of 10 μM to GAS Δ*emm1* mutant. A change of media color from red to yellow was interpreted as bacterial growth. Minimal bactericidal concentrations (MBC) were determined by plating 5 μL samples onto THA plates after 24 h of incubation to determine bactericidal histone concentrations.

### Histone H2A binding to bacteria

Bacteria at 10^7^ CFU/mL were incubated in RPMI with 5% THB in the presence of 31.25 μg/mL histone H2A for 30 min. Bacteria were incubated in PBS+10% BSA for blocking for 30 min. To determine histone binding, bacteria were incubated with anti-H2A antibody (1:500 dilution) for 30 min at 37 °C with agitation, followed by incubation with secondary Alexa488 antibody (1:1,000 dilution). Histone H2A binding to the bacterial surface was determined by flow cytometry. Bacteria were gated, and the fluorescence intensity was measured for 50,000 bacteria. Bacteria alone, bacteria incubated with histones and anti-histone H2A antibody and bacteria incubated with secondary antibody alone were used as negative controls. Flow cytometry data was analyzed using FlowJo v. 8.8.7.

### M1 protein pull-down with histones

Recombinant M1 (rM1) protein or truncated versions of the M1 protein, HVR+A (residues 42–132), A+B (residues 42–194) and B+C (residues 128–263) regions were used as bait and interaction with histone H2A was analyzed. A volume of 50 μL cobalt chelate resin was washed twice in binding buffer (5 mM imidazole, 100 mM NaCl, 20 mM Tris pH 7). A total of 5 μg rM1 protein were incubated with resin in binding buffer for 1 h at 37 °C. Samples were washed twice and 5 μg histone H2A was added for 1 h at 37 °C. After four serial wash steps with binding buffer, final elution was performed with 100 mM imidazole at 50 °C. The interaction of M1 protein with histones was determined by SDS-PAGE, followed by immunoblotting against histone H2A.

### Electron and confocal microscopic imaging of histones and bacteria

Bacteria at 1 × 10^9^ CFU/mL in RPMI+5% THB were incubated with histone H2A with rotation at 37 °C for 30 min for EM or for 3 h for confocal microscopy, respectively. For EM, bacteria were incubated with 31.25 μg/mL histone H2A, washed twice and fixed with 2% PFA followed by incubation with polyclonal, anti-histone H2A antibody (1:100 dilution). Secondary immunogold-labeled antibodies were used with 15 nm in size (1:50 dilution). Samples were visualized by EM at 13,000x, 23,000x and 49,000x magnification. Quantification of gold-particles was achieved by counting particles associated with >20 bacteria at 49,000x magnification. For confocal microscopy an established method was adapted^50^. Bacteria at 10^8^ CFU/mL were incubated with 62.5 or 150 μg/mL histones for 3 h at 37 °C. After incubation, bacteria were washed twice and subsequently washed serially in DPBS with 1 μg/mL FM4-64, then 2 μg/mL DAPI and 0.5 μM Sytox Green and analyzed by microcopy using ImageJ software v1.48f and CellProfiler 2.0. Sytox Green signal intensity was adjusted for WT and mutant bacteria samples in confocal microscopy images.

### Bacterial permeability assay

Bacteria were adjusted to OD_600_ = 0.2 in DPBS. Bacteria were incubated with histone H2A at concentrations ranging from 7.8 μg/mL to 1000 μg/mL in DPBS and Sytox Green at 5 μM. The fluorescence signal was quantified at 485 nm ex/527 nm em. Simultaneously, bacterial killing at designated histone H2A concentration and range of histone concentration was calculated by enumeration of CFU relative to initial inoculum.

### M1 protein model

The M1 protein model is based on the residues 42–194 representing the NT fragment of the protein sequence derived from GAS serotype M1 strain 5448 strain and was created using PyMol v. 1.7.4.4.

### Generation of truncated M1 protein lacking the N-terminus

GAS Δ*emm1*+pM1_ΔNT_ (residues 42–104, 190–453) was generated in pET28b from the construct of mature M1 protein (residues 42–453)^51^ using a QuickChange II Site-Directed mutagenesis kit according to manufacturer’s instructions. The deletion mutant was cloned into pDCerm expressing the *emm1* gene^32^ using sequence and ligation independent cloning. The plasmid was introduced into GAS Δ*emm1* mutant via electroporation and protein expression confirmed by western blot analysis.

### Statistical analysis

All data shown were collected from at least three independent experiments in triplicate, except microscopic analysis. Experiments using neutrophils were performed using cells isolated from a minimum of three different healthy volunteers. Data were combined and expressed as average ± SEM. Results were either analyzed by unpaired Student’s *t*-test or by the non-parametric Mann-Whitney test using GraphPad Prism version 7. *P* values < 0.05 were considered statistically significant.

## Additional Information

**How to cite this article**: Döhrmann, S. *et al*. Group A Streptococcal M1 Protein Provides Resistance against the Antimicrobial Activity of Histones. *Sci. Rep.* **7**, 43039; doi: 10.1038/srep43039 (2017).

**Publisher's note:** Springer Nature remains neutral with regard to jurisdictional claims in published maps and institutional affiliations.

## Supplementary Material

Supplementary Material
