# Supplementary material for: Group A Streptococcal M1 Protein Provides Resistance against the Antimicrobial Activity of Histones
Source: Sci Rep. 2017 Feb 21;7:43039. doi: 10.1038/srep43039 (PMC5318940; doi:10.1038/srep43039)
Supplement: Supplementary Material [file srep43039-s1.pdf]

Supplemental Information

Group A Streptococcal M1 Protein Provides Resistance against the Antimicrobial Activity of Histones

Simon Döhrmann[1], Christopher N. LaRock[1], Ericka L. Anderson[1], Jason N. Cole[1], Brinda Ryali[1], Chelsea Stewart[2], Poochit Nonejuie[3], Joe Pogliano[3], Ross Corriden[1,4], Partho Ghosh[2] and Victor Nizet[*1,5,6]

Contents:

Supplemental Materials and Methods
Figure S1
Figure S2
Figure S3
Figure S4
Table S1

Supplemental Materials and Methods

Histone H2A cytotoxicity against human cells

One day prior to the assay, 2 x 10^5 human epithelia A549 cells (ATCC CCL-185; USA) were seeded in 200 µL RPMI + 10 % fetal bovine serum (FBS; Life Technologies) and cultivated at 37 °C, 5 % CO_2. Prior to the assay, cells were washed once with DPBS. Either 2 x 10^5 neutrophils or A549 cells were seeded in a 96 well-plate in RPMI + 0.2 % BSA. Cytotoxicity was measured after incubating cells with 1, 10, 100 and 500 µg/mL histone H2A in 200 µL RPMI + 0.2 % BSA for 1 h at 37 °C, 5 % CO_2. Dead cells were stained with 1.5 µM propidium iodide (PI) (Life Technologies) and incubated for 5 min prior to analysis. Cells were imaged via fluorescence microscopy imaging using a Zeiss Observer D1 microscope with 20x objective and cell death was quantified by flow cytometry at FL-2 channel. Flow cytometry data was analyzed using FlowJo v. 8.8.7.

M1 protein expression on the GAS cell surface

M1 protein expression on GAS WT and Δ*emm1* mutant bacteria was analyzed by incubation of bacteria with mouse anti-M1 serum or naïve serum (1:100 dilution) for 30 min followed by goat anti-mouse Alexa 488 (Life Technologies) for analysis of a

total of 50,000 bacteria in Fl-1 channel using flow cytometry. For EM, log-phase bacteria were washed twice and fixed with 2 % PFA followed by incubation with mouse anti-M1 antiserum (1:100 dilution). Secondary goat anti-mouse immunogold-labeled antibodies of 10 nm size were used (1:50 dilution). Fixed samples were visualized using a Tecnai G2 Spirit BioTWIN transmission electron microscope. Random images were taken at 13,000 x, 23,000 x and 49,000 x with an Eagle 4 k HS digital camera.

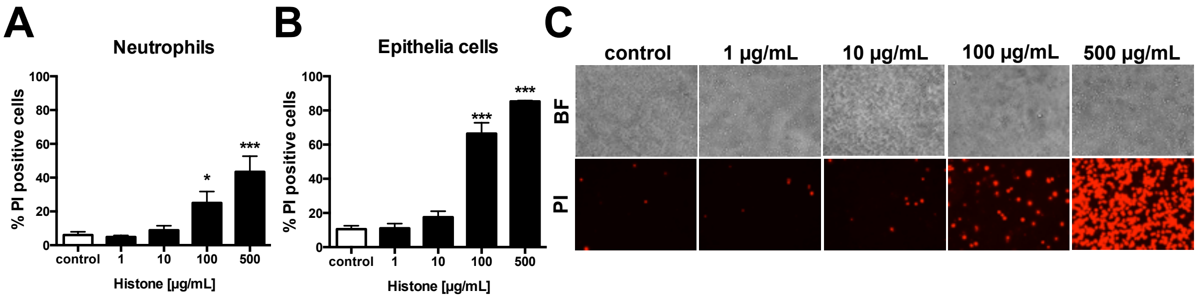

Figure S1. Histone-induced cytotoxicity at 1, 10, 100 and 500 µg/mL histone H2A determined by PI against (A) neutrophils and (B) A549 cells quantified using flow cytometry. (C) Visualization of histone-induced cytotoxicity in A549 cells by PI staining with fluorescent microscopy at 20x magnification. Results shown represent average ± SEM values and were analyzed by Student's *t*-test (n.s., not significant, *P<0.05 and ***P<0.001).

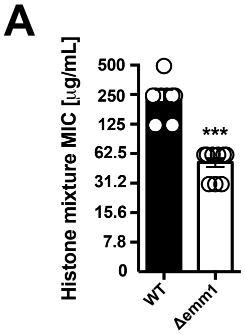

Figure S2. (A) MIC assay of GAS WT and Δ*emm1* mutant against histone mixture.

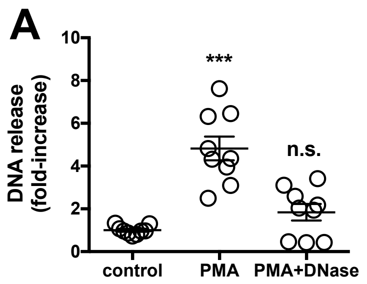

Figure S3. (A) Degradation of PMA-induced NETs with 100 U/mL DNase for 30 min after 3.5 h. Result shown represent average ± SEM values and were analyzed by Student’s *t*-test (n.s., not significant and ***P<0.001).

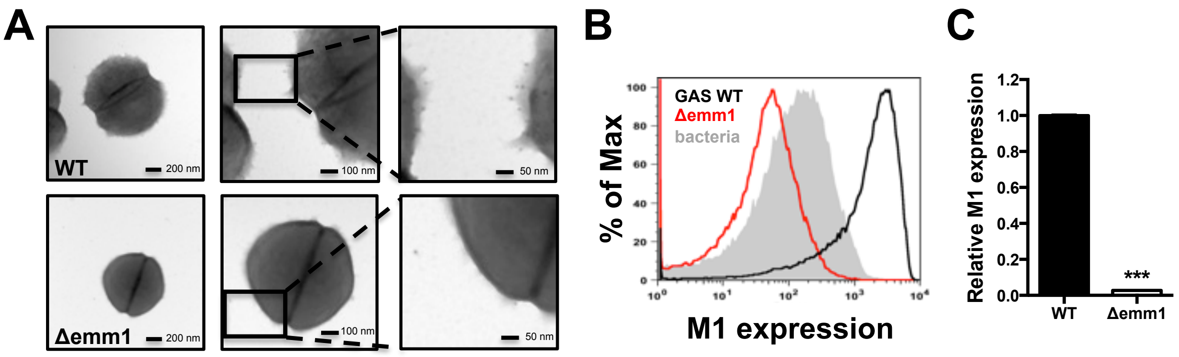

Figure S4. (A) M1 protein expression on GAS WT and Δ*emm1* mutant was analyzed by detection of M1 protein on bacterial surface with mouse anti-M1 serum followed by anti-mouse immunogold particles of 10 nm size and visualized by TEM at 11,000 and 23,000 and 49,000x magnification. (B) M1 protein expression on GAS WT and Δ*emm1* mutant was analyzed by flow cytometry and quantified relative to the fluorescent signal for GAS WT. Result shown in (B) represents average ± SEM values and was analyzed by Student's *t*-test (***P<0.001).

Supplementary Table 1. CFU counts in antibacterial experiments. CFU counts were listed in comparison to % survival of bacterial killing assay.

Figure	sample ID	mean surviving CFU	SD	% survival vs. inoculum
2D	GAS	4570.666667	1362.90314	90.20351
	MRSA	1836.605682	360.4460788	37.47276333
3E	GAS WT 1 h	1946666.667	485669.6408	94.35071333
	GAS WT 2 h	1575555.556	429537.8654	81.14296
	GAS WT 3 h	1391111.111	492682.5663	63.55810556
	Δemm1 1 h	1367777.778	1081316.071	28.72291667
	Δemm1 2 h	505555.5556	302411.6032	11.95432444
	Δemm1 3 h	198888.8889	135595.3949	9.511582222
3F	GAS WT 7.8 µg/mL	2377777.778	668539.0373	100
	GAS WT 15.6 µg/mL	2466666.667	482182.538	100
	GAS WT 32.1 µg/mL	2422222.222	399304.9517	100
	GAS WT 62.5 µg/mL	2188888.889	365528.5367	99.29676222
	GAS WT 125 µg/mL	1522222.222	705137.8904	75.93925667
	GAS WT 250 µg/mL	1397777.778	470817.8464	73.32338444
	GAS WT 500 µg/mL	1108888.889	305468.6745	70.73791444
	GAS WT 1000 µg/mL	1181111.111	483953.625	61.77463
	Δemm1 7.8 µg/mL	1644444.444	519882.4653	100
	Δemm1 15.6 µg/mL	1628888.889	705344.6754	99.17459778
	Δemm1 31.2 µg/mL	1677777.778	777460.2526	94.60317444
	Δemm1 62.5 µg/mL	881111.1111	669242.9388	87.16276
	Δemm1 125 µg/mL	520000	322529.0685	64.54842111
	Δemm1 250 µg/mL	444444.4444	115337.6685	34.13562444
	Δemm1 500 µg/mL	391111.1111	77208.23215	30.32377111
	Δemm1 1000 µg/mL	190000	143961.8005	16.24008556
4A	GAS WT	34044.44444	7365.987902	86.57496556
	Δemm1	18395.43829	2733.455904	47.96854111
	Δemm1+M1	28130.2015	5991.37245	74.29271889
4B	GAS WT	33866.66667	7515.317691	76.83277333
	Δemm1	24117.11998	5817.181713	67.68311222
	Δemm1+M1	39563.12918	18553.77091	113.9521
4C	GAS WT	30400	4270.831301	85.88306333
	Δemm1	26523.78092	6099.130126	75.33934
	Δemm1+M1	42368.82239	11815.51156	115.68812
4D	GAS WT	59644.44444	27788.08698	95.46187778
	GAS WT+anti-M1	38577.77778	17402.4264	62.32847
